# P-800. High prevalence of treatment initiation delays among people diagnosed with multidrug-resistant tuberculosis-Dushanbe, Tajikistan—2019-2021

**DOI:** 10.1093/ofid/ofae631.992

**Published:** 2025-01-29

**Authors:** Zulfiya H Tilloeva, Roberta Horth, Azamdjon Mirzoev, Rajabali Sharifov, Dilyara Nabirova, Bobochon Pirmahmadzoda

**Affiliations:** City Disinfection Station, Dushanbe, Republic of Tajikistan, Dushanbe, Dushanbe, Tajikistan; US Centers for Disease Control and Prevention, Dulles, Virginia; State Institution “Research Institute of Preventive Medicine of Tajikistan”, Dushanbe, Dushanbe, Tajikistan; Central Asia Region FETP, Dushanbe, Dushanbe, Tajikistan; CDC Central Asia office, Almaty, Almaty, Kazakhstan; City Center for Protection of the Population against Tuberculosis, Dushanbe, Republic of Tajikistan, Dushanbe, Dushanbe, Tajikistan

## Abstract

**Background:**

Multidrug resistant TB (MDR-TB) incidence is increasing in Central Asia, especially in Tajikistan, which is among the top 30 high-burden countries. Delays in treatment initiation are associated with increased transmission and poor health outcomes. In Dushanbe, the capital, the numbers and characteristics of people experiencing treatment delays are unknown. This information was needed to improve treatment programs and reduce transmission.

**Table 1.**
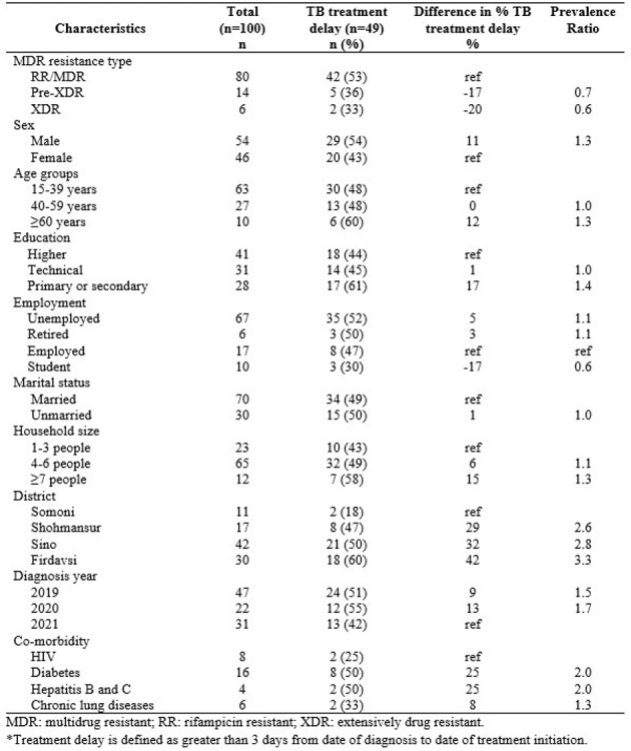
Characteristics of people with confirmed pulmonary MDR-TB experiencing treatment delays, Dushanbe, Tajikistan, 2019-2021

**Methods:**

We conducted a cross-sectional study of all persons, ages ≥15 years old, diagnosed with pulmonary MDR-TB in 2019-2021. We abstracted data in 2022 from the national electronic TB database OpenMRS. Treatment delay was defined as >3 days from MDR-TB diagnosis to treatment initiation. MDR-TB confirmation was based on molecular tests (including line probe assay and GeneXpert) and phenotypic drug sensitivity tests. We calculated treatment delay percentage by group and percent difference and prevalence ratio; statistical testing is not applicable in our study, which captures the entire population.

**Results:**

Of 100 people diagnosed with MDR-TB in 2019-2021, 6% had extensively drug-resistant (XDR) and 14% pre-XDR TB (Table 1). Also, 16% had diabetes, 8% had HIV, and 4% had hepatitis B and C. The median number of days from MDR-TB diagnosis to treatment initiation was 3 days (IQR: 1-7 days, Max: 225 days). Half (49%) experienced treatment delays. Proportion with delay was highest in 2020 (55%), the 1^st^ year of the COVID-19 pandemic. Also, 2 of 6 people with XDR-TB, had delayed treatment. The proportion experiencing delay was 11% greater for males than females, 12% greater for older populations than younger, 15% greater for larger households ( >7 people) compared to smaller (3 or less), and 17% greater for those primary/secondary compared university education. There was a 42% difference in the proportion of patients with delays between the district with the highest and lowest proportion of delay (60% and 18%, respectively).

**Conclusion:**

Half of people diagnosed with MDR-TB had treatment delays, including people with XDR-TB. Our findings highlight the need for immediate interventions that reduce time from diagnosis to treatment initiation, especially in districts and population groups with highest proportion of delayed treatment.

**Disclosures:**

**All Authors**: No reported disclosures

